# Food Insecurity is Associated with Poor Diet Quality Among Pregnant Adolescents and Adolescent Mothers in Ghana

**DOI:** 10.1016/j.cdnut.2025.107469

**Published:** 2025-05-19

**Authors:** Harriet Okronipa, Isabelle Posey, Christiana Nsiah-Asamoah, Moses K Klevor, Emmanuel Ayifah

**Affiliations:** 1 Department of Nutritional Sciences, Oklahoma State University, United States; 2 Department of Clinical Nutrition and Dietetics, University of Cape Coast, Ghana; 3 Department of Health Policy, Planning and Management, School of Public Health, University of Ghana, Ghana; 4 African Institute for Development Research and Evaluation

**Keywords:** diet quality, food insecurity, pregnant adolescents, adolescent mothers, Ghana

## Abstract

**Background:**

Food insecurity is a global public health problem and a likely determinant of poor diet quality. However, the relationship between food insecurity and diet quality among adolescents remains under-researched.

**Objective:**

This study examined the association between food insecurity and diet quality among pregnant adolescents and adolescent mothers in Ghana.

**Methods:**

A cross-sectional survey was conducted among pregnant adolescents (n = 216) and adolescent mothers (n = 206) aged 12-19 y in Cape Coast, Ghana. Diet Quality indicators, including Food Group Diversity Score (FGDS), Minimum Diet Diversity for women (MDD-W), ALL-5 indicator, and dietary factors for noncommunicable diseases (NCD-Protect and NCD-Risk) were assessed using the Global Diet Quality Questionnaire (DQQ). Food Insecurity was measured using the Child Food Insecurity Experience Scale (CFIES). Linear and logistic regression were used to examine the association between food insecurity and diet quality indicators, adjusting for relevant covariates.

**Results:**

Participants had a mean age of 18 ± 1.2 y. Most were out of school (70.50%) and unemployed (82.0%). Participants consumed 5 out of 10 food groups (FGDS 4.9 ± 1.7), and 56.8% met the MDD-W. The mean CFIES score was 9.5 ± 6.4, with 87.2% reporting some food insecurity in the past 30 d. Food insecurity was significantly associated with FGDS and MDD-W but not the ALL-5 indicator. Participants who experienced food insecurity were less likely to meet the MDD-W (odds ratio: 0.39; 95% confidence interval: 0.20, 0.77), consume NCD protective foods (β ± SE = −0.59 ± 0.22, *P* = 0.0078) and consume foods linked to NCD risk (−0.74 ± 0.26, *P* = 0.004).

**Conclusion:**

Food insecurity was significantly associated with poor diet quality among pregnant adolescents and adolescent mothers in Ghana. Interventions addressing food insecurity may improve diet quality in this population.

## Introduction

The period of adolescence is an important stage of the life cycle, characterized by rapid growth and development. During this period, adolescents begin to establish their independence and make their own food choices. The dietary habits they develop may be long-lasting and impact their health in adulthood [1]. Adequate nutrition during this period is therefore important to help support growth and achieve full developmental potential. This need is even more critical among the subpopulation of pregnant adolescents and adolescent mothers due to the increased demands of pregnancy and lactation [2,3]. Despite the importance of this life stage, they have not received much attention [4]. Designing interventions to improve nutrition among this group requires an understanding of the unique challenges they face regarding making healthy dietary choices for themselves and their children, including food insecurity.

Food insecurity, defined as the lack of regular access to enough safe and nutritious food for normal growth and development and an active and healthy life, remains an important public health problem [5]. According to estimates from the Food and Agricultural Organization (FAO), 2.4 billion people in the world (29.6% of the global population) were moderately or severely food insecure in 2022, with the highest rates recorded in low-income economies, including Africa [5]. In Ghana, almost half of the population (49.1%) experienced food insecurity in 2022 [6]. These high rates are concerning, particularly among adolescent pregnant girls, given the negative impact of food insecurity on several birth outcomes.

Although food insecurity has been extensively studied, there remains a paucity of research that specifically examines its association with diet quality among pregnant adolescents and adolescent mothers in Ghana. Most existing studies on food insecurity in Ghana focus on broader populations, neglecting the unique vulnerabilities of pregnant girls and adolescent mothers. Furthermore, the available research often emphasizes food security experiences of adolescents in the general population or school-going age adolescents [[7], [8], [9]]. This lack of focus limits our understanding of how food insecurity directly influences this vulnerable population's dietary behaviors, diet quality, and nutritional outcomes.

Among the general population, people with inadequate and inconsistent access to food may be more likely to consume cheap, energy dense, and nutrient-poor diets and highly processed foods and snacks and less likely to consume foods considered to be healthy such as fruits, vegetables, whole grains [[10], [11], [12]]. A study conducted in Kenya and Uganda found that in highly food-insecure communities, calorie deficiencies are prevalent, and 40 to 50% of women do not reach their recommended minimum dietary quality thresholds [13].

Adequate diets during pregnancy and lactation are also important for the growing fetus and for the child. Inadequate dietary intake during pregnancy has been reported to be associated with stillbirth, premature birth, low birth weight, and maternal and prenatal death [14]. Food insecurity may impact diet quality during this period. However, in the case of adolescent girls, pregnancy adds an extra layer of demand for essential nutrients to support fetal growth and maternal health. Food insecurity during this period of pregnancy among adolescent girls can compromise diet quality, leading to deficiencies in key nutrients like iron, calcium, folate, and protein, which are vital for pregnancy outcomes and adolescent development. Studies have revealed that food insecurity during adolescence, which results in poor diet quality, is associated with increased risk of anemia, low prepregnancy weight, increased stress, anxiety, and depression, all of which adversely affect maternal and neonatal health outcomes [[15], [16], [17], [18]]. Other negative effects of poor diet quality as a result of food insecurity during adolescence and pregnancy are low birth weight, preterm birth, and impaired cognitive and physical development in children [19,20]. This perpetuates health disparities and limits future opportunities for both the adolescent mother and her child.

Despite the importance of nutrition during pregnancy and adolescence, empirical data on diet quality and food insecurity prevalence among pregnant adolescents and adolescent mothers remain limited, particularly in low-and middle-income countries such as Ghana. Additionally, although some studies have explored maternal nutrition in Ghana, they often overlook the intersection of adolescence, pregnancy, and food insecurity. The distinct challenges faced by pregnant adolescents, such as social stigma, reduced educational attainment, and limited access to healthcare, compound risks associated with poor diet quality. In the current study, we aim to describe diet quality and food insecurity among pregnant adolescents and adolescent mothers in Ghana and to examine if they are related. This study is justified by the urgent need to generate context-specific evidence to guide interventions aimed at improving diet quality and food security for pregnant adolescents and adolescent mothers in Ghana. By identifying the association between food insecurity and diet quality, this research contributes to filling a critical knowledge gap and provides a basis for designing targeted nutritional intervention programs to improve dietary habits, nutritional outcomes, and overall health for pregnant adolescents, adolescent mothers, and their children. The findings will also support policymakers in developing integrated strategies that address food insecurity and its detrimental effects on adolescent girls in Ghana.

## Methods

### Study setting and participants

This study was conducted in Cape Coast in the Central Region of Ghana between July and December 2022 as part of the Healthy Adolescent Nutrition in Ghana (HANIG) project. This region, according to the 2014 Ghana Demographic Health Survey, had the highest proportion (7%) of adolescents pregnant with their first child and the second highest proportion of adolescents who have begun childbearing in the country [21].

Potential participants were recruited from 32 communities (16 urban, 16 rural) in and around Cape Coast using door-to-door strategies and snowball sampling techniques and from community health centers. Within each community, community guides helped identify pregnant adolescents and adolescent mothers. Once potential participants were identified, they were visited at home by field workers and informed about the study. Potential participants were screened for eligibility using a screening questionnaire. Participants were eligible to participate in the study if they were *1*) 12 – 19 y of age, *2*) pregnant or had a child aged 5 y or younger, and *3*) not suffering from any chronic illness that will affect their participation in the study. Participants who were deemed eligible were taken through an informed consent process and enrolled in the study if they agreed to be part of the study and gave their consent by means of a thumbprint. For participants aged <18 y, informed consent was obtained from the parent or caregiver, and assent was obtained from the child. Once enrolled, participants were scheduled for data collection at the study center. Ethical approval for the study was obtained from the Institutional Review Boards of Oklahoma State University and the University of Cape Coast.

### Data collection

All data collection activities took place at a central location within each community, usually at the community center. Data were collected by trained field workers (with a bachelor’s degree) through in-person interviews using questionnaires translated into the local language (Twi/Fanti). Data was collected electronically on tablets using the Kobo Toolbox software.

#### Diet Quality

Data on diet quality was collected using the Global Diet Quality Questionnaire (DQQ) for Ghana [22], developed by the Global Diet Quality project (dietquality.org/countries/gha). This simple and rapid-assessment tool consists of food items that correspond to 29 food groups and measures several aspects of diet quality. Participants were asked to indicate “yes or no” to whether they have consumed sentinel food items within each food group in the past 24 h. Using information from the DQQ and a guide developed by the Global Diet Quality project (dietquality.org/tools), we constructed diet quality indicators which included indicators of dietary adequacy (Food Group Diversity Score, FGDS; Minimum Dietary Diversity for women, MDD-W; ALL-5 indicator) and dietary factors associated with noncommunicable disease (NCD) risk (NCD-Protect, NCD-Risk) [22].

##### FGDS and MDD-W

These indicators were calculated based on 10 food groups comprising *1*) grains, plantains, roots and tubers (such as cassava, yam); *2*) pulses (such as beans, bambara beans); *3*) nuts and seeds (such as groundnuts, cashews); 4) milk and milk products; *5*) meat, poultry, and fish; *6*) eggs; *7*) dark green leafy vegetables (such as cocoyam leaves, amaranth leaves); *8*) other vitamin A–rich fruits and vegetables (such as carrot, mango); *9*) other vegetables (such as tomatoes, garden eggs); and *10*) other fruits (such as orange, banana). The FGDS, a semicontinuous variable, was generated by summing up scores for each of the 10 food groups consumed and ranged from 0 (consumed none of the food groups) to 10 (consumed all 10 food groups). The MDD-W was defined as the proportion of women who consumed ≥5 out of the 10 food groups. Both the MDD-W and FGDS are indicators of micronutrient adequacy [22].

##### ALL-5 indicator

This variable is an indicator of micronutrient adequacy and reflects the consumption of diets that include five food groups often recommended in national guidelines around the world, including starchy staples, fruits, vegetables, animal-source foods, pulses, nuts or seeds [22].

##### NCD-Protect

This variable was generated based on 9 food groups comprising *1*) whole grains; *2*) pulses; *3*) nuts and seeds; *4*) vitamin A–rich orange vegetables; *5*) dark green leafy vegetables; *6*) other vegetables; *7*) vitamin A–rich fruits; *8*) citrus; and *9*) other fruits. The NCD-Protect variable, which ranges from 0 to 9, was calculated by summing up scores for each of the 9 food groups consumed. A higher score indicates the inclusion of more health-promoting foods in the diet that protect against NCDs [22].

##### NCD-Risk

This variable consists of 8 food groups, namely *1*) soft drinks and sodas *2*) baked/grain-based sweets, *3*) other sweets, *4*) processed meat (double weighted), *5*) unprocessed red meat; *6*) deep fried food; *7*) fast food and instant noodles; and *8*) packaged ultra processed salty snacks. The NCD-Risk variable was calculated by summing up scores for each of the 8 food groups consumed and ranged from 0 to 9 (processed meat is double weighted). The higher the score, the lower the likelihood of meeting global recommendations on dietary risk factors of NCDs [22].

#### Food Insecurity

Food insecurity data were collected using the Child Food Insecurity Experience Scale (CFIES), a 10-item scale that asks about a child’s food insecurity experiences in the month preceding the interview [23]. Response options for each item on the questionnaire range from 0 to 2 (0 = never, 1 = 1 or 2 times, 2 = many times). For each participant, responses on all 10 items were summed to obtain a total CFIES score ranging from 0 to 20, with higher scores indicating more food insecurity experiences. The total CFIES scores were then used to categorize participants as having “no food insecurity experiences” (score of 0), “few food insecurity experiences” (score 1–6), "several food insecurity experiences" (score 7–10) or "many food insecurity experiences" (score 11–20). We also created binary categories of food insecurity: “food insecure” (few/several/many food insecurity experiences) compared with “food secure” (no food insecurity experience).

#### Other measures

Sociodemographic data, including age, pregnancy status, level of education, current enrollment in school, employment status, number of children, and living arrangements, were also collected. Anthropometric measurements were taken by trained field workers: Height was measured to the nearest 0.1 cm using a stadiometer (Seca 217); weight was measured to the nearest 0.1 kg using a weighing scale (Seca 874). Height and weight measurements were taken in duplicates. A third measurement was taken if the values differed by >0.5 cm and 0.1 kg for height and weight, respectively.

### Statistical analysis

Sociodemographic characteristics, diet quality indicators, and food insecurity were summarized using means and standard deviation for continuous variables or frequencies and percentages for categorical variables.

We examined the association between each of the 5 diet quality indicators and adolescent food insecurity. For the continuous indicators FGDS, NCD-Protect, and NCD-Risk outcomes, we tested associations with food insecurity as continuous and binary (food insecure compared with food secure) predictors using linear regression. For the binary diet quality indicators MDD-W and ALL-5, we used logistic regression analysis. All regressions were modeled unadjusted and adjusted for prespecified covariates. In adjusted analysis, we controlled for prespecified sociodemographic variables, including adolescent age, pregnancy status, highest level of education attained, current enrollment in school, employment status, BMI, number of children aged <5 y, and living arrangement, if they were significantly associated with the outcome at 20% level of significance [24]. The results of logistic regression analysis were reported as odds ratio (AOR) along with the 95% confidence interval (CI). We set the level of significance (α) at 0.05 for all analyses and considered an association to be statistically significant if *P* < 0.05. All analyses were conducted using SAS, version 9.4 (Cary).

## Results

We screened 441 participants, 8 of whom were ineligible due to their age. Of the 433 eligible participants, 11 did not show up for enrollment. A total of 422 were enrolled and participated in data collection activities. Of these, 51% (n = 216) were pregnant adolescents and 48.8% (n = 206) were adolescent mothers.

Sociodemographic characteristics of study participants are shown in Table 1. The mean age of participants was ∼18 years. About a quarter were currently enrolled in school, and less than a fifth (18.9%) reported having some form of employment. The majority (63%) lived with their parents; only 3% lived alone.

**Table 1:** Sociodemographic characteristics of pregnant adolescents and adolescent mothers who participated in the Healthy Adolescent Nutrition in Ghana (HANIG) study.

Characteristic	Overall	Pregnant adolescents *n* = 216	Adolescent mothers *n* = 206
	*N* = 422		
Age (y), [mean ± SD]	17.8 ± 1.2	17.7 ± 1.4	17.9 ± 1.1
BMI scores (kg/m^2^) [mean ± SD]	22.3 ± 3.5	22.9 ± 3.4	21.7 ± 3.5
Currently enrolled in school [(*n* (%)]	103 (24.5)	65 (30.1)	38 (18.4)
Level of education [(*n* (%)]			
None	7 (1.7)	5 (2.3)	2 (0.9)
Primary	95 (22.6)	53 (24.5)	42 (20.4)
Junior High School	258 (61.4)	123 (56.9)	137 (66.5)
Senior High School	60 (14.3)	35 (16.2)	25 (12.1)
Living status [(*n* (%)]			
Lives alone	13 (3.11)	8 (3.7)	5 (2.4)
Lives with parents	264 (63.2)	134 (62.6)	130 (63.4)
Other^1^	141 (33.7)	72 (33.6)	70 (34.1)
Currently employed [(*n* (%)]	79 (18.9)	38 (17.8)	41 (20.0)

1 Lives with other people including relatives, friends

### Food insecurity

**FIGURE 1:**
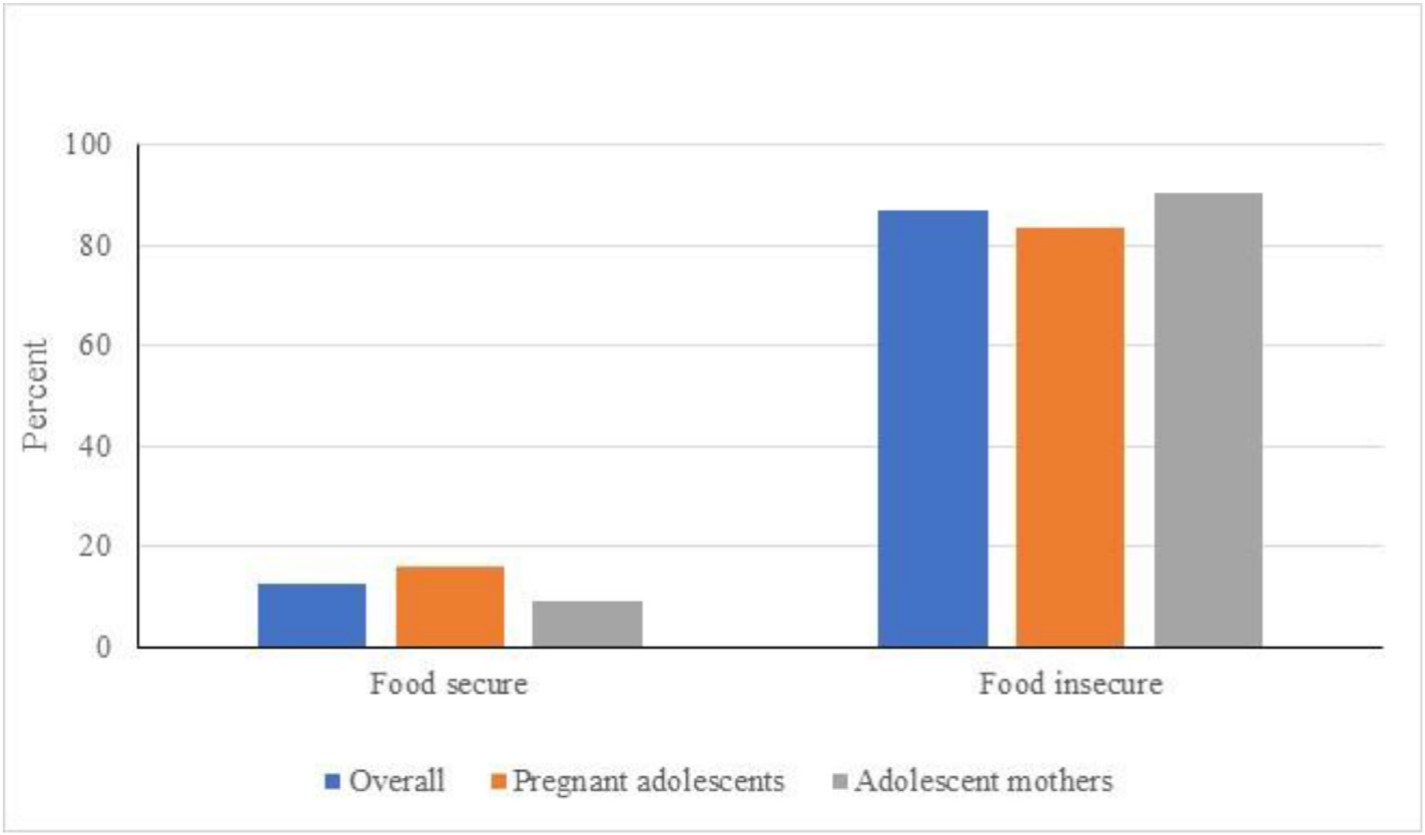
Proportions of adolescents reporting food insecurity experiences Food insecurity experiences were measured using the 10-item Child Food Insecurity Experience Scale with response options ranging from 0 to 2 (0 = never, 1 = 1 or 2 times, 2 = many times). Summed response scores range from 0-20 (0, no food insecurity experiences; 1-6, few food insecurity experiences; 7-10, several food insecurity experiences and 11-20, many food insecurity experiences). Prevalence of food insecurity (few/several/many) was significantly higher among adolescent mothers compared to pregnant adolescents (P=0.0331).

The mean ± SD CFIES score was 9.5 ± 6.4 out of a total score of 20. Overall, ∼4 out of every 5 participants (87.2%) reported ≥1 experience with food insecurity in the last month. Of these, about two-fifths (42%) reported having many food insecurity experiences in the last month. Figure 1 shows the prevalence of food insecurity among subgroups: prevalence was significantly higher among adolescent mothers compared with pregnant adolescents (83.7% compared with 90.7%, *P* = 0.0331).

### Dietary quality

#### Nutrient adequacy

**FIGURE 2:**
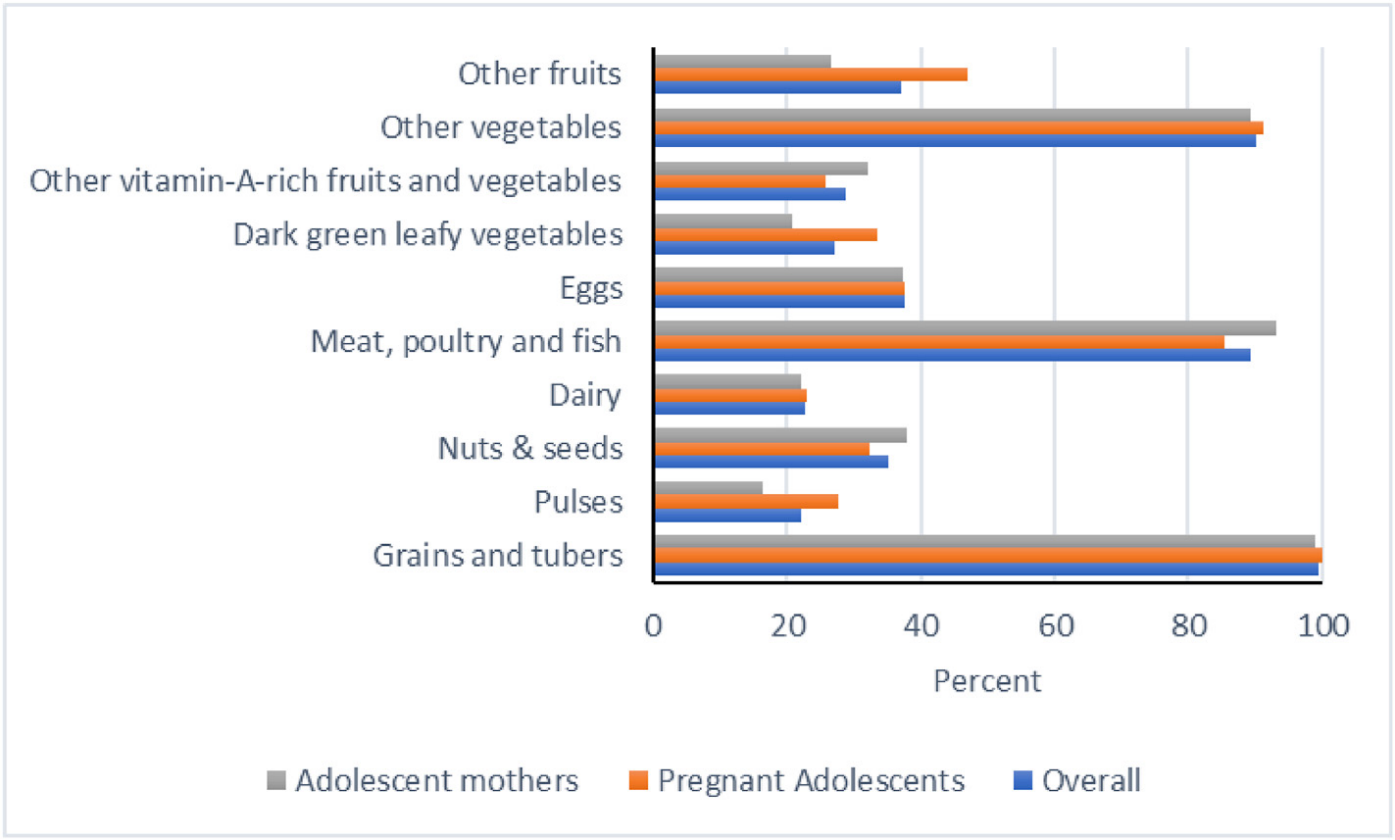
Proportions of adolescents reporting consumption of different food groups Diet quality was measured using the Global Diet Quality Questionnaire for Ghana [22]. The 10 food groups comprise of 1) grains, plantains, roots &, tubers (such as cassava, yam), plantains 2) pulses (such as beans, bambara beans); 3) nuts and seeds (such as groundnuts, cashews); 4) milk and milk products; 5) meat, poultry, and fish; 6) eggs; 7) dark green leafy vegetables (such as cocoyam leaves, amaranth leaves); 8) other vitamin A–rich fruits and vegetables (such as carrot, mango); 9) other vegetables (such as tomatoes, garden eggs); and 10) other fruits (such as orange, banana).

Table 2 shows diet quality indicators for our study participants, including indicators for nutrient adequacy. The mean ± SD FGDS was 4.9 ± 1.7, which means that participants consumed ∼5 out of 10 recommended food groups. About 56.9% of participants met the MDD-W, implying that 43.1% did not meet the MDD-W. Figure 2 reflects the food groups included in the MDD-W calculations. The consumption of pulses (22.1%), nuts and seeds (35.1%), and other fruits (37.0%) were less common **(**Figure 2**).** However, grains and tubers (99.5%), other vegetables (90.1%), and meat, poultry, and fish (94.5%) were commonly consumed by most of the study participants. Only about a quarter (23.4%) consumed all 5 recommended food groups. Nutrient adequacy indicators were similar between pregnant adolescents and adolescent mothers, except that adolescent mothers were less likely than pregnant adolescents to consume ALL-5 recommended food groups (*P* = 0.04, Table 2).

**TABLE 2:** Diet Quality Indicators among pregnant adolescents and adolescent mothers who participated in the Healthy Adolescent Nutrition in Ghana (HANIG) study.

Diet Quality Indicator	Overall	Pregnant adolescents	Adolescent mothers	*P* value
FGDS, mean ± SD^1^	4.9 ± 1.7	5.0 ± 1.6	4.7 ± 1.7	0.0762
MDD-W, *n* (%)^2^	236 (56.9)	128 (60.1)	108 (53.5)	0.1879
ALL-5, *n* (%)^3^	97 (23.4)	59 (27.7)	38 (18.8)	0.0424
NCD-Protect, mean ± SD^4^	3.2 ± 1.5	3.4 ± 1.5	3.0 ± 1.4	0.002
NCD-Risk, mean ± SD^5^	2.1 ± 1.8	1.9 ± 1.7	2.3 ± 1.8	0.0198

1 FGDS, Food Group Diversity Score, based on 10 food groups comprising 1) grains, plantains, roots &, tubers (such as cassava, yam); *2*) pulses (such as beans, bambara beans); *3*) nuts and seeds (such as groundnuts, cashews); 4) milk and milk products; *5*) meat, poultry, and fish; *6*) eggs; *7*) dark green leafy vegetables (such as cocoyam leaves, amaranth leaves); *8*) other vitamin A–rich fruits and vegetables (such as carrot, mango); *9*) other vegetables such as tomatoes, garden eggs); and *10*) other fruits (such as orange, banana).

2 MDD-W, Minimum Dietary Diversity for Women, consumption of ≥5 out of 10 food groups.

3 ALL-5, Consumption of diets that include 5 food groups often recommended in national guidelines around the world. Food groups include 1) starchy staples 2) fruits 3) vegetables 4) animal-source foods and 5) pulses, nuts or seeds.

4 NCD-Protect, Consumption of food groups that protect against noncommunicable diseases including *1*) whole grains (such as maize-based meals – kenkey, banku); *2*) pulses (such as beans, bambara beans); *3*) nuts and seeds (such as groundnuts, cashews); *4*) vitamin A–rich orange vegetables (such as carrots, sweet potato that are orange inside); *5*) dark green leafy vegetables; *6*) other vegetables; *7*) vitamin A–rich fruits (such as mango, papaya); *8*) citrus (such as orange, tangerine); and *9*) other fruits.

5 NCD-Risk, Consumption of food groups that increase risk for noncommunicable diseases including 1) soft drinks and sodas; *2*) baked/grain-based sweets (such as cakes, rock buns); *3*) other sweets (such as toffee, chocolate); *4*) processed meat (such as sausage, corned beef; double weighted); *5*) unprocessed red meat (such as beef, goat); *6*) deep fried food (such as fried yam, fried potato); *7*) fast food and instant noodles (such as Indomie noodles, pizza); and *8*) packaged ultra processed salty snacks (such as packaged yellow plantain chips, Pringles).

#### NCD protection and risk

**FIGURE 3:**
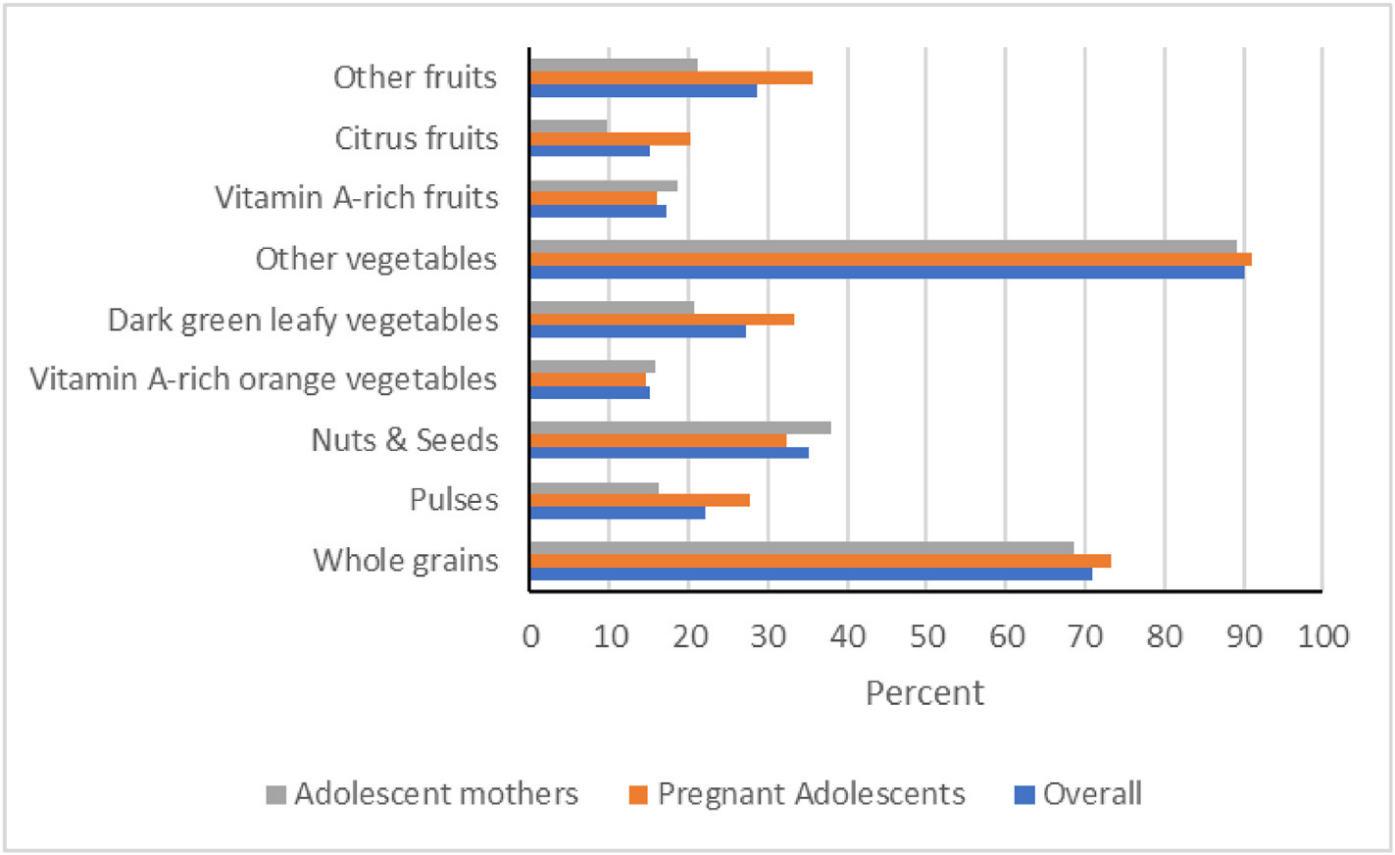
Proportions of adolescents reporting consumption of foods that protect against noncommunicable diseases (NCD-Protect). NCD-Protect outcome includes 9 food groups comprising 1) whole grains (such as maize-based foods - kenkey, banku); 2) pulses (such as beans, bambara beans); 3) nuts and seeds (such as groundnuts, cashews); 4) vitamin A–rich orange vegetables (such as carrot, sweet potato that are orange inside); 5) dark green leafy vegetables (such as cocoyam leaves, amaranth leaves); 6) other vegetables (such as tomatoes, garden eggs); 7) vitamin A–rich fruits (such as mango, papaya); 8) citrus (such as orange, tangerine); and 9) other fruits (such as orange, banana).

**FIGURE 4:**
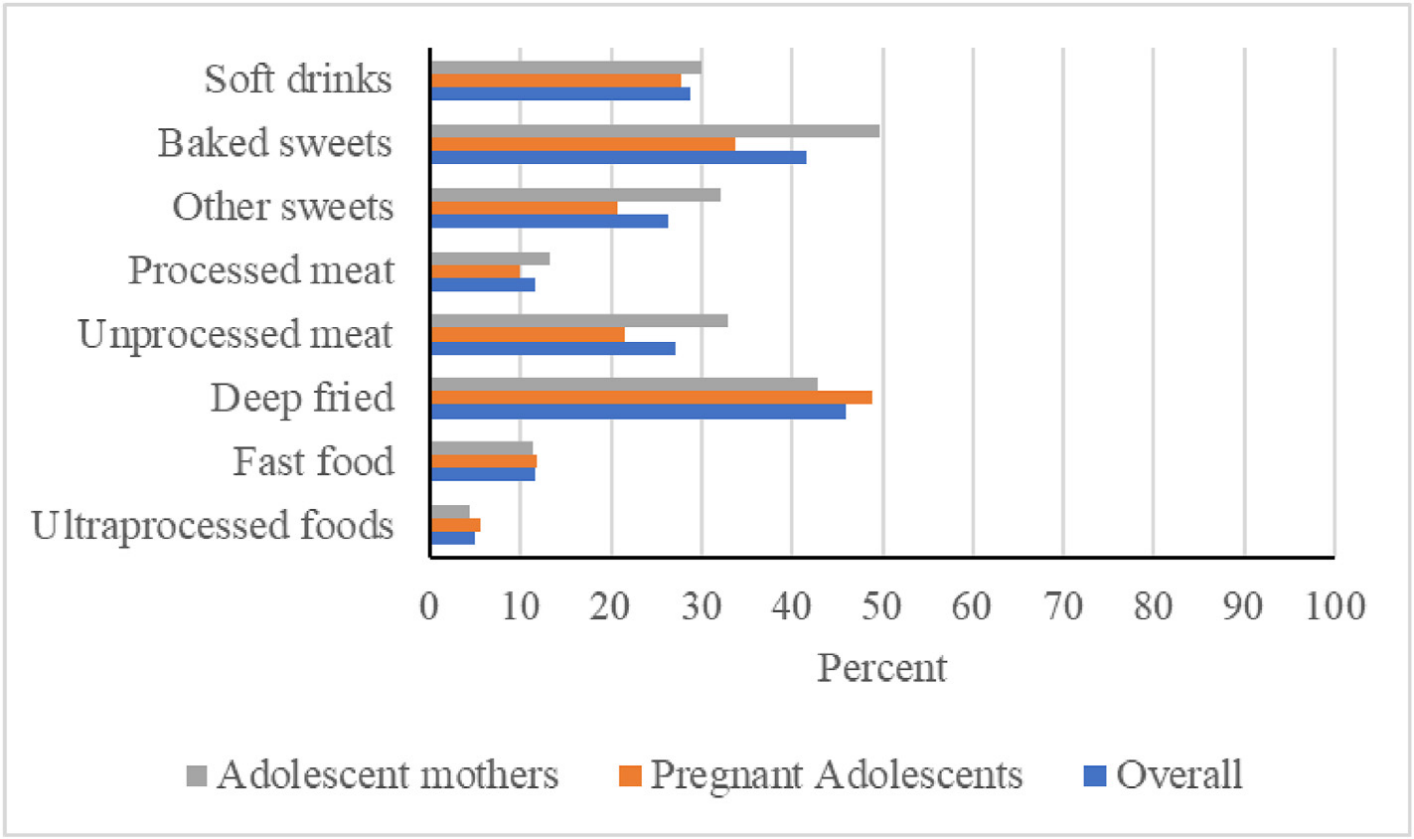
Proportions of adolescents reporting consumption of foods that increase risk of noncommunicable diseases (NCD-Risk). NCD-Risk outcome includes 8 food groups comprising 1) soft drinks and sodas 2) baked/grain-based sweets (such as cakes, rock bun); 3) other sweets (such as toffee, chocolate); 4) processed meat (such as sausage, corned beef); 5) unprocessed red meat (such as beef, goat); 6) deep fried food (such as fried yam, fried potato); 7) fast food and instant noodles (such as Indomie noodles, pizza); and 8) packaged ultraprocessed salty snacks (such as packaged yellow plantain chips, Pringles).

Table 2, Figure 3, and Figure 4 show the consumption of foods that protect or increase risk for NCDs, respectively. Whole grains and other vegetables were commonly consumed by more than two-thirds of adolescents (Figure 3). The consumption of foods that increased risk for NCDs, including deep fried foods (45.9%) and baked sweets (41.6%), was moderate, whereas processed meat (11.3%), fast food (11.3%) and ultra processed foods (5.1%) were less commonly consumed (Figure 4). Pregnant adolescents compared to adolescent mothers, were more likely to consume foods that are protective against NCDs and less likely to consume foods that increase risk for NCDs (Table 2).

### Association between food insecurity and diet quality

Table 3 and Table 4 present the adjusted and unadjusted results for the association between diet quality indicators and food insecurity. We observed a significant association between food insecurity (continuous and binary variables) and dietary adequacy indicators FGDS and MDD-W but not ALL-5 (Table 3, TABLE 4) in both unadjusted and adjusted analyses. The odds of meeting the MDD-W were 0.39 times lower among adolescents who reported an experience with food insecurity (few, several, or many) compared with those who had no experience with food insecurity (AOR 0.39, 95% CI: 0.20, 0.77, Table 3).

**Table 3:** Association between food insecurity and diet quality (indicators of dietary adequacy) among pregnant adolescents and adolescent mothers who participated in the Healthy Adolescent Nutrition in Ghana (HANIG) study.

	FGDS^1^		MDD-W^2^		ALL-5^3^	
	Unadjusted Estimate ± SE *P* value	Adjusted^4^ Estimate ± SE (*P* value)	Unadjusted OR (95%CI) (*P* value)	Adjusted^4^ OR (95%CI) (*P* value)	Unadjusted OR (95%CI) (*P* value)	Adjusted^4^ OR (95%CI) (*P* value)
Food insecurity score^5^	−0.04 ± 0.01 (0.0005)	−0.04 ± 0.01 (0.0006)	0.95 (0.92, 0.98) 0.0008	0.95 (0.92, 0.98) 0.0013	0.98 (0.94, 1.01) 0.2602	0.98 (0.95, 1.02) 0.3485
Food Insecurity status^6^						
Food insecure	−0.88 ± 0.24 0.0003	−0.85 ± 0.24 0.0005	0.38 (0.20, 0.73) 0.0040	0.39 (0.20, 0.77) 0.0060	0.66 (0.35, 1.26) 0.2099	0.71 (0.37, 1.35) 0.2973
Food secure	Ref	Ref	Ref	Ref	Ref	Ref

1 FGDS, Food Group Diversity Score, based on 10 food groups comprising 1) grains, plantains, roots &, tubers (such as cassava, yam); *2*) pulses (such as beans, bambara beans); *3*) nuts and seeds (such as groundnuts, cashews); 4) milk and milk products; *5*) meat, poultry, and fish; *6*) eggs; *7*) dark green leafy vegetables (such as cocoyam leaves, amaranth leaves); *8*) other vitamin A–rich fruits and vegetables (such as carrot, mango); *9*) other vegetables such as tomatoes, garden eggs); and *10*) other fruits (such as orange, banana).

2 MDD-W, Minimum Dietary Diversity for women, consumption of ≥5 out of 10 food groups.

3 ALL-5, Consumption of diets that include 5 food groups often recommended in national guidelines around the world. Food groups include 1) starchy staples 2) fruits 3) vegetables 4) animal-source foods and 5) pulses, nuts or seeds.

4 Adjusted for pregnancy status and number of children.

5 Summation of responses on the 10-items on the Child Food Insecurity Experience Scale.

6 Food insecure (few/several/many food insecurity experiences) compared with food secure (no food insecurity experiences).

**TABLE 4:** Association between food insecurity and diet quality (NCD-associated factors) among pregnant adolescents and adolescent mothers who participated in the Healthy Adolescent Nutrition in Ghana (HANIG) study.

	NCD-Protect^1^		NCD-Risk^2^	
	Unadjusted Estimate ± SE *P* value	Adjusted^3^ Estimate ± SE (*P* value)	Unadjusted Estimate ± SE *P* value	Adjusted^4^ Estimate ± SE (*P* value)
Food insecurity score^5^	−0.03 ± 0.01 (0.0121)	−0.03 ± 0.01 (0.0272)	−0.00 ± 0.01 (0.7209)	−0.01 ± 0.01 (0.3597)
Food Insecurity status^6^				
Food insecure	−0.65 ± 0.22 (0.0030)	−0.59 ± 0.22 (0.0078)	−0.65 ± 0.26 (0.0119)	−0.74 ± 0.26 (0.0039)
Food secure	Ref	Ref	Ref	Ref

1 NCD-Protect, Consumption of food groups that protect against noncommunicable diseases including *1*) whole grains (such as maize-based meals – kenkey, banku); *2*) pulses (such as beans, bambara beans); *3*) nuts and seeds (such as groundnuts, cashews); *4*) vitamin A–rich orange vegetables (such as carrots, sweet potato that are orange inside); *5*) dark green leafy vegetables; *6*) other vegetables; *7*) vitamin A–rich fruits (such as mango, papaya); *8*) citrus (such as orange, tangerine); and *9*) other fruits.

2 NCD-Risk, Consumption of food groups increases risk for noncommunicable diseases. The food groups include *1*) soft drinks and sodas *2*) baked/grain-based sweets (such as cakes, rock buns); *3*) other sweets (such as toffee, chocolate); *4*) processed meat (such as sausage, corned beef; double weighted); *5*) unprocessed red meat (such as beef, goat); *6*) deep fried food (such as fried yam, fried potato); *7*) fast food and instant noodles (such as Indomie noodles, pizza); and *8*) packaged ultraprocessed salty snacks (such as packaged yellow plantain chips, Pringles).

3 Adjusted for pregnancy status and number of children.

4 Adjusted for pregnancy status and number of children and current employment status.

5 Summation of responses on the 10-items on the Child Food Insecurity Experience Scale.

6 Food insecure (few/several/many food insecurity experiences) compared with food secure (no food insecurity experiences).

Similarly, both continuous and binary food insecurity variables were negatively associated with dietary factors protective of NCDs in both adjusted and unadjusted analyses (Table 4). For example, adolescents who reported food insecurity experiences had lower NCD-Protect (β ± SE = −0.59 ± 0.22, *P* = 0.0078) scores compared with adolescents who had no food insecurity experiences. However, food insecurity was negatively associated with NCD-Risk scores, although it was only significant for the binary but not the continuous food insecurity variable (Table 4).

## Discussion

In this study, we described the food insecurity experiences of pregnant adolescents and adolescent mothers and how these experiences relate to their diet quality. We found that majority of pregnant adolescents and adolescent mothers in our study had experienced some level of food insecurity over the previous month. Diet quality was inadequate overall, and food insecurity was negatively associated with diet quality indicators.

We found high rates of food insecurity (87%) among this population of pregnant adolescents and adolescent mothers, with adolescent mothers more likely than pregnant adolescents to report food insecurity, suggesting a potential exacerbation of vulnerability due to the added responsibilities and economic demands of caring for a child. According to the Ghana Statistical Service (GSS), 49.1% (regional variation 27.2% to 80.3%) of the population in Ghana experienced food insecurity in 2022 [6]. Although our ability to compare our results with current literature is limited due to lack of available data specific to this subpopulation of pregnant adolescent and adolescent mothers, we can compare our results with similar populations, including nonpregnant adolescent girls and pregnant adult women. Our findings of high food insecurity rates are similar to studies in Ghana that examined household food insecurity among adolescents [17,18,23,25]. In a study conducted by Frongillo et al. [23] to validate the CFIES measure among a sample of nonpregnant adolescents aged 10 to 17 y in Northern Ghana, 79.4% of adolescents reported having any experience with food insecurity. Other studies conducted in Ghana have also reported adolescent food insecurity rates of ≤70% [17,18,25]. Although the levels of food insecurity reported in the present study are slightly higher than reported in previous studies, it is understandable, given the added responsibility of pregnancy or having to take care of a child. As highlighted in qualitative studies, pregnant adolescents and adolescent mothers may face stigma and isolation from their families and community and may be less likely to have support in providing for themselves and their families, sometimes even resulting in suicidal behavior risks [[26], [27], [28]]. That said, it is also likely that the differences could be due to how food insecurity data was collected. Although the above-mentioned studies obtained food insecurity information by interviewing caregivers of adolescents, in the present study, adolescents directly responded to questions about their own food insecurity experiences. Studies have shown that caregivers may incorrectly believe they are shielding their children from food insecurity experiences, resulting in a lower than actual reporting of food insecurity rates when mothers respond on behalf of their children [29].

Second, our results suggest that diet quality was suboptimal among our study sample of pregnant adolescents and adolescent mothers. The FAO recommends that all women achieve the MDD-W, which reflects micronutrient adequacy, an important dimension of diet quality. In our study, we found that ∼40% of pregnant adolescents and 47% of adolescent mothers did not meet the recommended MDD-W of consuming ≥5 out of the 10 recommended food groups in a day. Additionally, only ∼20% consumed all 5 recommended food groups (ALL-5 indicator). This finding underscores the dual burden of malnutrition common in low-and middle-income countries like Ghana, where inadequate access to diverse foods often results in diets dominated by monotonous staple foods with low micronutrient density [30].

Our results align with a study in Ghana that found that 56% of pregnant adolescents did not meet the MDD-W recommendations [31]. These results align with the MDD-W reports for the general Ghanaian population, as reported by the Global Diet Quality project [32]. They found that among Ghanaians aged >15 y, only 44% met MDD-W and 20% met ALL-5. Our results, however, differ slightly compared with other studies among pregnant adolescents and pregnant adults in other parts of the African continent. Among pregnant adolescents in Ethiopia, for instance, as many as 78% did not meet the MDD-W recommendation [33]. In studies conducted among adult pregnant women, rates ranging from 39% in Kenya [34] to 75% in South Africa [35] have been reported. Differences in results may be due to country differences in other risk factors, including food insecurity, demographic, and socioeconomic factors.

Interestingly, adolescents scored low on both the NCD-Protect (3.2 out of 9) and NCD-Risk (2.1 out of 9) indicators, indicating low consumption of foods that protect against NCDs as well as low consumption of foods that increase risk of NCDs. These scores are somewhat similar to the general Ghanaian population (NCD-Protect, 3.0 out of 9; NCD-Risk, 1.4 out of 9) [32]. Reduced access to these foods may explain the low rates observed overall. The low scores on NCD-Risk suggest that adolescents in the study also consumed low amounts of unhealthy processed foods, which might reflect limited market access or purchasing power rather than health-conscious choices. In other words, although low NCD-Risk scores which suggest limited intake of processed foods, sugary beverages, and high-fat diets seem beneficial and commendable, they may instead indicate a lack of overall dietary variety rather than a conscious avoidance of unhealthy foods in this population of pregnant adolescents and adolescent mothers. These findings are consistent with studies indicating that populations experiencing food-insecurity often consume monotonous diets as a coping mechanism, which consequently results in a failure to meet nutritional requirements [36,37].

Third, we found that food insecurity was negatively associated with diet quality indicators, including FGDS, MDD-W, NCD-Protect, and NCD-Risk. For instance, the odds of meeting the MDD-W were 0.39 times lower among adolescents who had any experience with food insecurity compared with those who had no experience with food insecurity. This finding could provide evidence of the typical coping mechanisms of food insecurity in our study population, where diet diversity is compromised and there is low consumption of foods that both promote and protect against NCDs, respectively. This trend is understandable given that typical foods that increase risk for NCDs, such as fast food, are expensive. Food insecurity may decrease the consumption of these foods due to limited access to economic resources.

Although the literature that examines the relationship between food insecurity and diet quality among pregnant adolescents and adolescent mothers is limited, our findings agree with the trend found in current research. Pregnant adolescents in the Ashanti region of Ghana were found to have higher odds of inadequate dietary diversity when they experienced hunger [30]. Among pregnant women and adult mothers, decreased diet diversity was associated with food insecurity [38,39]. This relationship could be due to the effects of limited financial and food resources decreasing the opportunity to consume a varied diet.

The findings of this study have several policy and programmatic implications. This includes the recommendation to address the high prevalence of food insecurity by exploring social protection programs such as cash transfers, food vouchers, and in-kind food assistance, which should be tailored to the needs of pregnant adolescents and adolescent mothers. These interventions can help increase access to diverse and nutrient-rich foods.

Again, the study findings have implications for nutrition education and behavior changes among this vulnerable group of adolescent girls by integrating nutrition education into maternal and child health programs in order to improve awareness of dietary diversity and the importance of consuming NCD- protective foods. Adolescent girls should be empowered with knowledge about low-cost, locally available, and nutrient-dense food options. Further research is needed to understand the specific barriers and facilitators of dietary improvement among pregnant adolescents and adolescent mothers. Longitudinal studies are recommended to provide insights into the long-term impacts of food insecurity and inadequate diet quality on maternal and child health outcomes. Additionally, monitoring and evaluation frameworks should be established to assess the effectiveness of interventions aimed at reducing food insecurity and improving diet quality of pregnant adolescents and adolescent mothers.

Our study has multiple strengths. We used a novel tool, the DQQ, that allowed us to measure protective and unhealthy aspects of diets. This tool has been validated for use in our Ghanaian population, has low respondent burden, and is currently being used in various countries to measure multiple key population-level diet quality indicators. Additionally, data were collected by trained field workers who understood the local language, reducing variability in data collection and reducing cultural biases. Despite these strengths, a few limitations should be considered. First, unlike other tools that use the open-recall method, the DQQ is a list-based tool that asks about specific foods within each food group. It is possible that participants consumed foods that may not have been listed in the food group, but that could have contributed to their total food intake. However, it is unlikely that this impacted our results because the DQQ is designed to include foods that are most consumed within each country and specific to that country [22]. Second, the cross-sectional nature of our study limits the ability to make any causal inferences between food insecurity and diet quality.

The findings of this study contribute to the limited understanding of food insecurity experiences and diet quality among pregnant adolescents and adolescent mothers in Ghana. Results suggest that this population experiences high rates of food insecurity and has suboptimal diet quality. Findings also provide evidence of significant association between food insecurity experiences and several diet quality indicators such as diet diversity, food group adequacy, and consumption of foods protective against NCDs that promote general health and well-being. By improving food security and diet quality, it is possible to enhance maternal and child health outcomes, reduce risk of NCDs, and break cycles of poverty and malnutrition in this vulnerable population.

## Author contributions

The authors’ responsibilities were as follows – HO, CNA, MKK, and EA designed research; HO, CNA, and MKK conducted research; HO analyzed the data; and HO and IP wrote the paper. HO had primary responsibility for final content. All authors reviewed and approved the final manuscript.

## Data availability

Data described in the manuscript, code book, and analytic code will be made available upon request, pending application and approval.

## Funding

This project was supported by Faculty [HO] Start-up Funds from the Office of the Vice President for Research, Oklahoma State University. The content is solely the responsibility of the authors and does not necessarily represent the official views of the funder. The sponsors were not involved in any component of the report and provided no restrictions regarding publication.

## Conflict of interest

The authors report no conflicts of interest.
